# Biochemical Properties and Anti-Biofilm Activity of Chitosan-Immobilized Papain

**DOI:** 10.3390/md19040197

**Published:** 2021-03-31

**Authors:** Diana R. Baidamshina, Victoria A. Koroleva, Svetlana S. Olshannikova, Elena Yu. Trizna, Mikhail I. Bogachev, Valeriy G. Artyukhov, Marina G. Holyavka, Airat R. Kayumov

**Affiliations:** 1 Laboratory of Molecular Genetics of Microorganisms, Kazan (Volga Region) Federal University, Kazan 420008, Russia; prosto-di@mail.ru (D.R.B.); trizna91@mail.ru (E.Y.T.); 2 Department of Biophysics and Biotechnology, Voronezh State University, Voronezh 394018, Russia; koroleva_victoria@bk.ru (V.A.K.); Olshannikovas@gmail.com (S.S.O.); artyukhov@bio.vsu.ru (V.G.A.); holyavka@rambler.ru (M.G.H.); 3 Biomedical Engineering Research Centre, St. Petersburg Electrotechnical University, St. Petersburg 197376, Russia; rogex@yandex.com; 4 Interdepartment Research Laboratory, Kazan State Academy of Veterinary Medicine named after N.E. Bauman, Kazan 420029, Russia

**Keywords:** adsorption immobilization, papain, chitosan, bacterial biofilms

## Abstract

Chitosan, the product of chitin deacetylation, is an excellent candidate for enzyme immobilization purposes. Here we demonstrate that papain, an endolytic cysteine protease (EC: 3.4.22.2) from *Carica papaya* latex immobilized on the matrixes of medium molecular (200 kDa) and high molecular (350 kDa) weight chitosans exhibits anti-biofilm activity and increases the antimicrobials efficiency against biofilm-embedded bacteria. Immobilization in glycine buffer (pH 9.0) allowed adsorption up to 30% of the total protein (mg g chitosan^−1^) and specific activity (U mg protein^−1^), leading to the preservation of more than 90% of the initial total activity (U mL^−1^). While optimal pH and temperature of the immobilized papain did not change, the immobilized enzyme exhibited elevated thermal stability and 6–7-fold longer half-life time in comparison with the soluble papain. While one-half of the total enzyme dissociates from both carriers in 24 h, this property could be used for wound-dressing materials design with dosed release of the enzyme to overcome the relatively high cytotoxicity of soluble papain. Our results indicate that both soluble and immobilized papain efficiently destroy biofilms formed by *Staphylococcus aureus* and *Staphylococcus epidermidis*. As a consequence, papain, both soluble and immobilized on medium molecular weight chitosan, is capable of potentiating the efficacy of antimicrobials against biofilm-embedded *Staphylococci*. Thus, papain immobilized on medium molecular weight chitosan appears a presumably beneficial agent for outer wound treatment for biofilms destruction, increasing antimicrobial treatment effectiveness.

## 1. Introduction

Proteolytic enzymes are widely used for various medical purposes such as debridement, as well as removal of necrotic and infected tissues in wounds or burns. Plant proteases exhibit some common advantages compared with mammalian ones because of lower risks of disease transmission [1,2]. Among them, papain (EC 3.4.22.2), an endolytic cysteine protease enzyme from the papaya (*Carica papaya* L.) latex, is nowadays offered as an anti-inflammatory, anti-coagulant, and hemolytic agent, also facilitating debridement and speeding up tissue recovery [3,4,5,6]. In addition, papain was reported as an anti-biofilm, anti-plaque, and anti-gingivitis agent [7,8,9,10,11,12].

Topical application of enzymes for wounds treatment is often limited by their low stability during storage due to autolysis. These limitations can be generally overcome by the enzymes’ immobilization on various insoluble carriers [13,14,15]. Additional advantages of enzyme immobilization include the enhancement of their biocatalytic properties, thereby increasing their stability and reusability [16,17,18]. The immobilized biocatalytic complexes provide an attractive basis for the development of novel therapeutic tools for the treatment of both old and newly emerging pathologies. Compared with other enzymes, immobilization of proteases is generally limited by high molecular weight of proteins, the substrates for proteases that in turn limits the number of active enzyme molecules to those with the active center being oriented to the medium. On the other hand, immobilized proteases become protected from the auto-proteolysis, this way increasing their stability during both storage and application [13,19,20]. 

Multiple attempts have been made to stabilize the papain, including covalent immobilization, interaction with metal ions, copolymerization with glutaraldehyde, immobilization in agarose, covalent binding with polyethersulfone, modification with succinic anhydride, simple adsorption in Celite^®^, ion absorption, adsorption on nylon-based immobilized copper ion affinity membrane [21], adsorption on electrospun nylon nanofiber as affinity membrane [22], inclusion in starch-based gel, and incorporation in nitrile fiber enriched with amino groups, as well as immobilization on the surface of cotton fabric [23], sepharose, nanoparticles, incorporation in niosomes, nanospheres, liposomes, and many others [23,24,25,26,27,28,29,30,31,32,33]. In some of the latest assays, both antibacterial and anti-biofilm activities of immobilized papain have been reported [6,34]. While the above approaches generally enhanced the protein stability and its optimal temperature, reduction of the specific activity and the enzymatic reaction speed of the immobilized enzyme compared with soluble papain appeared to be their common limitations.

Chitosan, the product of chitin diacetylation, is a universal sorbent that binds with a wide range of substances of both organic and inorganic origin, thus being an excellent candidate for enzyme immobilization purposes [35,36,37,38]. Low-cost, large-scale availability, antimicrobial activity, biodegradability, non-toxicity, and bio-adhesive properties make chitosan a promising carrier for many enzymes to obtain highly active and thermostable immobilized catalysts [12,39,40,41,42,43]. Moreover, its biocompatibility and non-toxicity make it a potential candidate for both conventional and novel drug delivery systems. Particularly, chitosan-based matrixes are of interest in tissue engineering for controlled drug release, as well as for tissue remodeling due to its fibrous and porous properties. Besides, chitosan is absolutely safe for humans and can be completely degraded after use, thereby obtaining environmentally friendly products [44,45,46]. 

Here we show that papain immobilized on a matrix of medium molecular (200 kDa) and high molecular (350 kDa) weight chitosans provides topical destruction of bacterial biofilms and increases the efficiency of biofilm-embedded bacteria treatment.

## 2. Results

### 2.1. Immobilization of Papain on Chitosan Matrixes

Immobilization of papain on the matrix of either medium molecular or high molecular weight chitosans was carried out by using the adsorption-from-solution approach. The largest amount of papain (mg per g of carrier) adsorbed on medium molecular weight chitosan was observed when using Tris-glycine buffer (pH 8.5–9.0), borate buffer supplemented with KCl in (pH 8.0–10.0), and glycine buffer (pH 8.6–10.5) (Table 1). The highest total and specific activities of immobilized enzyme were obtained after the protein adsorption from acetate buffer (pH 5.0–5.8), glycine buffer (pH 8.6–10.5), and Tris-glycine buffer (pH 8.5–9.0). 

In the case of high molecular weight chitosan, maximal protein adsorption was obtained in the borate buffer (pH 8.0–10.0) supplemented with KCl, Tris-glycine buffer (pH 8.5–9.0), and glycine buffer (pH 8.6–10.5) (Table 2). Highest total and specific activity of papain could be adsorbed in the acetate buffer (pH 5.0–5.8), glycine buffer (pH 8.6–10.5), Tris-glycine buffer (pH 8.5–9.0), and borate buffer supplemented with KCl (pH 8.0–10.0).

In summary, considering the protein content, total and specific activity, the 0.05 M glycine buffer pH 9.0 was proposed as an optimized solution for the immobilization of papain on the matrix of both medium and high molecular weight chitosans (Table 1 and Table 2), and this was the way the immobilized enzyme was further analyzed.

### 2.2. Catalytic Properties of the Native and Immobilized Papain

**Figure 1:**
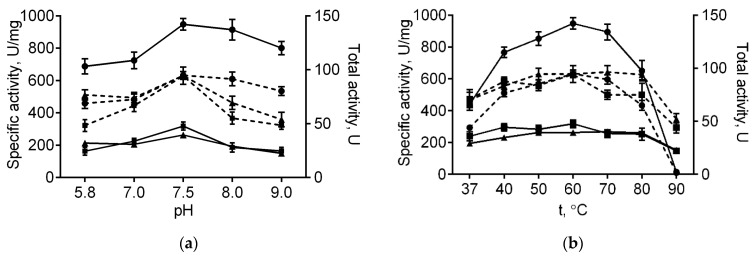
The pH (**a**) and temperature (**b**) dependencies of the total (dashed lines) and specific activities (solid lines) of the soluble papain (circles), as well as the enzyme immobilized on either medium molecular weight (squares) or high molecular weight chitosan (triangles).

The immobilized papain was further investigated in order to characterize its stability and catalytic properties in comparison with the soluble enzyme. Optimal temperature and pH for papain immobilized on either medium or high molecular weight chitosans were similar to those of the soluble enzyme (60 °C and 7.5, respectively, see Figure 1). With increasing pH, the total activity of the enzyme immobilized on both chitosans decreased compared with the soluble one, apparently, due to their reduced solubility at high pH values. Further, at 80 °C free enzymatic activity reduced by 68% of its initial value, while complete inactivation could be observed at 90 °C (Figure 1b). By contrast, the papain immobilized on either medium or high molecular weight chitosans retained 80% and 98% at 80 °C, as well as 45% and 57% of their initial activity at 90 °C, respectively (Figure 1b). Of note, neither total nor specific activities of the enzyme immobilized on both chitosans decreased at low temperatures. These data suggest that adsorption on chitosan alternates neither optimal temperature nor pH, while increasing the thermal stability of papain, apparently due to the restricted conformational flexibility of the protein, thereby preventing its premature denaturation.

**Figure 2:**
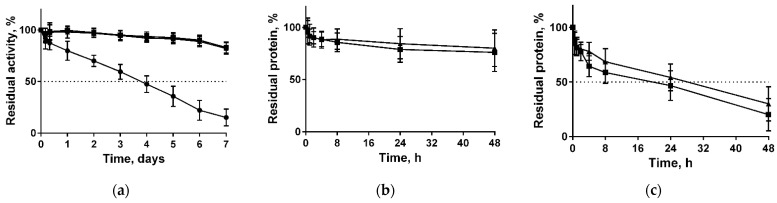
Enzymatic stability of soluble papain (circles) and enzyme immobilized on either medium molecular weight (squares) or high molecular weight chitosan (triangles) when stored at 37 °C in 50 mM Tris buffer pH 7.5 (**a**); desorption of papain immobilized on either medium molecular weight (squares) or high molecular weight chitosan (triangles) at 37 °C in either 50 mM Tris buffer pH 7.5 (**b**) or in 0.9% NaCl (**c**).

Long-term stability of both free and immobilized papain in various solutions were also tested. For that, both enzymes were incubated at 37 °C in 50 mM Tris buffer pH 7.5. The half-life of soluble papain was approximately three days, while the remaining activity of papain immobilized on both types of chitosans was about 93% and 83% of the initial values after three and seven days, respectively (Figure 2a). The extrapolated half-life times were found to be 78 ± 0.7 h, 520 ± 0.7 h, and 441 ± 1.4 h for soluble, immobilized on medium molecular weight chitosans, and immobilized on high molecular weight chitosans, respectively. Further, the stabilization factor (SF) (the half-life’s ratio of soluble and immobilized enzyme) was estimated as 6.67 for the medium and 5.65 for the high molecular weight chitosans, respectively.

Since the enzyme did not bind covalently to the carrier, one could expect the dissociation of the complex while being in solutions. The desorption of papain from both chitosans in 50 mM Tris-HCl pH 7.5 after 48 h of incubation did not exceed 24% (Figure 2b). By contrast, in physiological saline (0.9% NaCl) approximately 50% of protein dissociated into solution in 24 h, and 75% of protein was desorbed in 48 h (Figure 2c).

**Figure 3:**
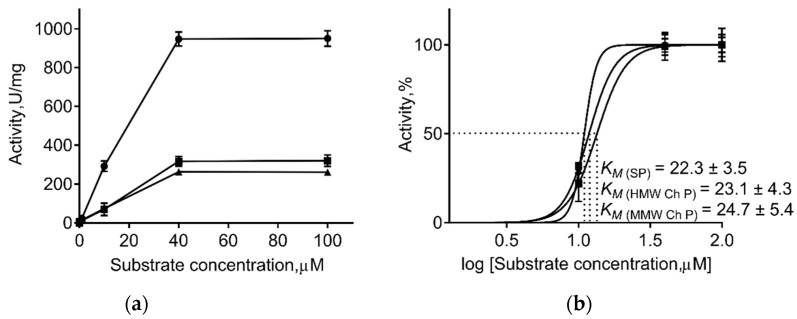
The effect of substrate concentration on activities of soluble papain (SP, circles) and enzyme immobilized on either medium molecular weight (MMW Ch P, squares) or high molecular weight chitosan (HMW Ch P, triangles) **(a**). Calculation of substrate concentration providing the half-maximal activity of enzymes (K_m_) (**b**).

Generally, immobilization of the enzyme on insoluble carriers changes the kinetic parameters of enzymatic catalysis. Therefore, the apparent maximum steady-state rate (V_max_), the apparent Michaelis constant (K_m_), and apparent catalytic constant (k_cat_) values of the immobilized and free papain were calculated (Table 3, Figure 3). As one can see from Table 3, immobilization on both chitosans did not change the K_m_ of papain, suggesting no changes in the enzymatic affinity to substrate. By contrast, a 3-fold decrease in V_max_ and 8–12-fold decrease in k_cat_ values was observed, suggesting the repression of the catalyst, apparently as the consequence of conformational restrictions due to immobilization.

### 2.3. Anti-Biofilm Properties of Immobilized Papain

**Figure 4:**
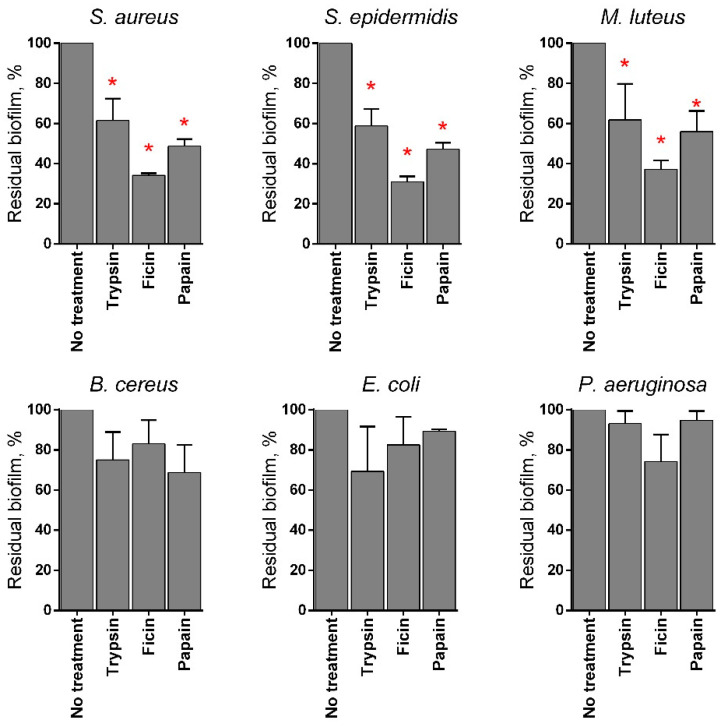
The effects of trypsin, ficin, and papain on bacterial biofilms. The 48 h old biofilms were gently washed by BM broth and loaded with fresh BM broth supplemented with trypsin, ficin, and papain at concentration of 1 mg·mL^−1^. After 24 h incubation, biofilms were assessed by crystal-violet staining. Asterisks (*) denote statistically significant difference between treated and untreated wells (*p* < 0.05).

**Figure 5:**
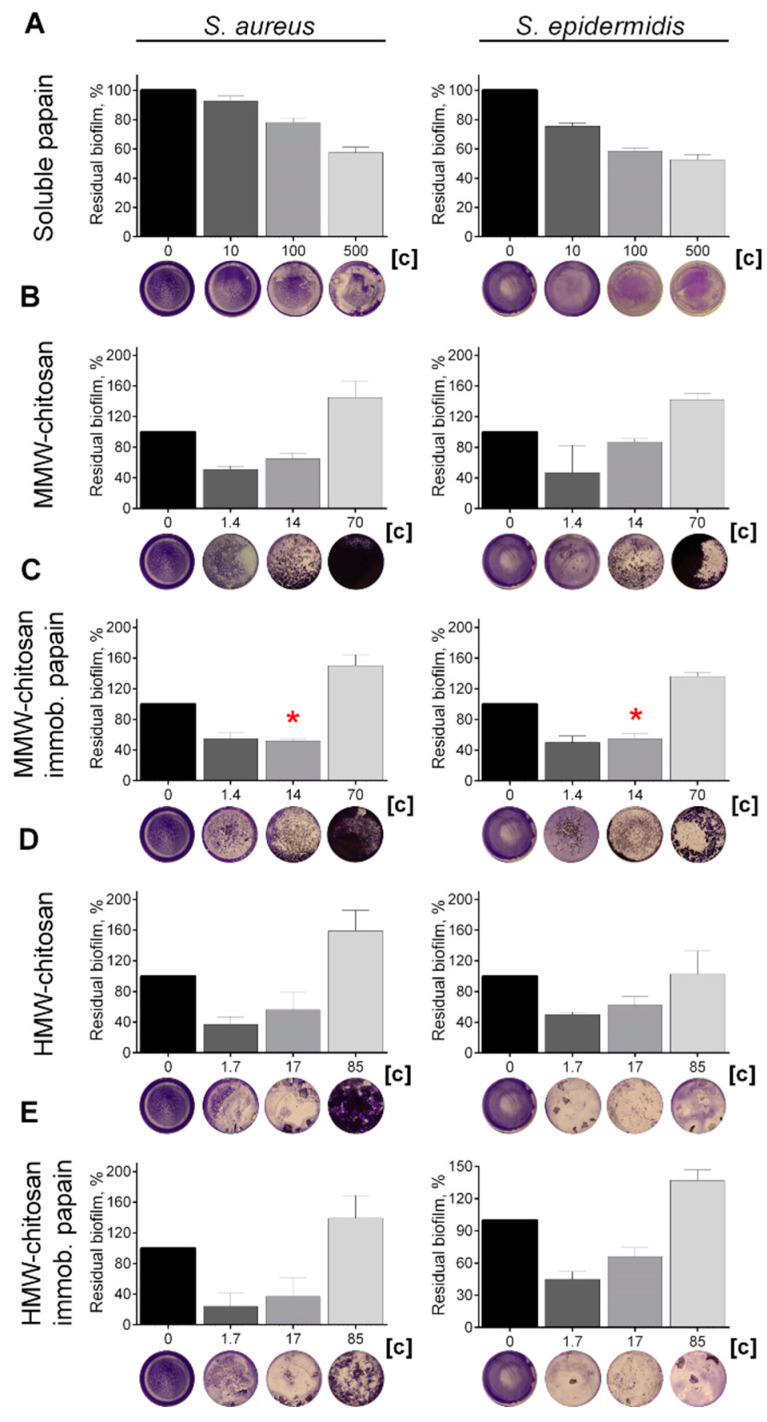
The effect of (**A**) soluble, (**C**) medium- and (**E**) high molecular weight chitosan-immobilized papain as well as solely (**B**) medium- and (**D**) high molecular weight chitosans on *S. aureus* and *S. epidermidis* biofilms. The 48 h old biofilms were gently washed by BM broth and loaded with fresh BM broth supplemented with substances as indicated. Soluble papain concentrations were 10, 100, or 500 μg·mL^−1^. Papain immobilized on medium molecular weight chitosan was added until final concentrations of 1.4, 14, or 70 mg·mL^−1^ (corresponding to the same final papain concentration by the total protein), respectively. Papain immobilized on high molecular weight chitosan was added at concentrations of 1.7, 17, or 85 mg·mL^−1^, respectively. Incubation was followed for 24 h and residual biofilms were quantified by crystal-violet staining. Asterisks (*) denote statistically significant difference between samples treated with either pure chitosan or chitosan with immobilized papain (*p* < 0.05). Untreated wells were considered as 100%.

Various proteases have been reported to disrupt microbial biofilms on wound surfaces [47,48] including the papain [9,11,12]. Therefore, the ability of chitosan-immobilized papain to disrupt the bacterial biofilms was assessed. For that, 48 h old biofilms were grown on 24-well plates and washed twice, followed by incubation in fresh BM broth supplemented by papain (1 mg mL^−1^) for 24 h, as previously indicated for immobilized ficin [49]. After 24 h, the residual biofilms were subjected to crystal violet staining. Trypsin, a protease that is commonly used for wound treatment [50], and ficin with confirmed anti-biofilm activity [51] served as references. Soluble papain destroyed biofilms formed by Staphylococci and *Micrococcus luteus*, although it appeared less efficient compared with ficin (See Figure 4). In contrast, neither for *Bacillus cereus* nor for the Gram-negative bacteria could a similar effect be observed. Taking into account the significant medical relevance of *Staphylococci* on topical wounds, the anti-biofilm activity of the immobilized papain was further tested on solely staphylococcal biofilms. The immobilized papain samples were added until final concentrations corresponded to soluble papain solutions with either 10, 100, and 500 µg protein mL^−1^, while the same amounts of chitosan alone were tested as a control (1.4, 14.0, and 70 mg mL^−1^ for the medium molecular weight chitosan and 1.7, 17.0, and 85.0 for the high molecular weight chitosan, respectively). Pure papain reduced the biofilm biomass of both *S. aureus* and *S. epidermidis* twice at the concentration of 500 µg protein mL^−1^. Both medium and high molecular weight chitosans themselves also decreased the biofilm biomass at low concentrations (1.4–17 mg mL^−1^), apparently due to the mechanical removal of the biofilm (Figure 5b,d). At high concentrations, chitosan clumps remained adherent to the plate surface, leading to the artificial increase of retained dye. Therefore, the decrease of the biofilm biomass in wells treated with immobilized papain was compared with the biofilms in wells treated with the same amount of pure chitosan (compare Figure 5b,c; Figure 5d,e). After treatment with medium molecular weight chitosan immobilized papain in amounts corresponding to 100 µg mL^−1^ of the protein, the biofilm biomass of both *Staphylococci* decreased by 25%, similar to the effect of the soluble enzyme (compare Figure 5a,c). Higher concentrations did not lead to the biofilm destruction, apparently because of the chitosan adhesion to the plate. Papain immobilized on high molecular weight chitosan exhibited similar effects only on *S. aureus*, while only moderate suppression of the *S. epidermidis* biofilm (which did not lead to statistically significant differences) could be observed.

### 2.4. Increasing the Efficiency of Antimicrobials Against Staphylococcal Biofilms by Soluble and Citosan-Immobilized Papain

While embedded into the matrix of the biofilm, bacterial cells become inaccessible to both antibiotics and biocides. The above data suggest that chitosan-immobilized papain could increase the efficiency of antimicrobials against biofilm-embedded bacteria by destroying the biofilm structure, as had been shown previously in other model investigations [48,49]. To test the above hypothesis, next 48 h old biofilms were incubated for 24 h in the presence of papain (either soluble or immobilized on chitosans with the final protein concentration of 100 µg protein mL^−1^) and antimicrobials at their respective 4× MBCs (minimal bactericidal concentrations, see Table 4 for values). After incubation, the plates were washed twice by sterile PBS to remove residual planktonic and detached cells and quantified by counting CFUs (see Section 5. Materials and Methods).

**Figure 6:**
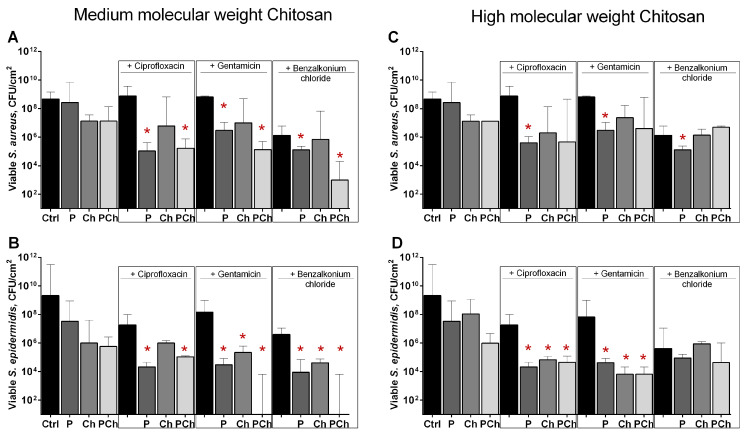
The effect of soluble (P), (**A**,**B**) medium- and (**C**,**D**) high molecular weight chitosan-immobilized papain (PCh) as well as solely (**A**,**B**) medium- and (**C**,**D**) high molecular weight chitosans (Ch) on susceptibility of biofilms-embedded *S. aureus* and *S. epidermidis* to antimicrobials. Soluble or immobilized on either medium or high molecular weight chitosan papain was added to 48 h old biofilms until final concentrations of 500 µg·mL^−1^, 70 mg·mL^−1^, 85 mg·mL^−1^, respectively (similar total protein). Chitosans were added until 70 mg·mL^−1^ and 85 mg·mL^−1^, respectively. Ciprofloxacin, gentamicin, or benzalkonium chloride were added up to final concentrations of 8× MBC (see Table 4 for values). After 24 h incubation, the biofilms were washed twice with sterile 0.9% NaCl. The adherent cells were scratched, resuspended, and their viability was analyzed by CFUs count. Asterisks (*) denote statistically significant difference of residual CFUs in control wells (solely antimicrobials) and wells with combined treatment (*p* < 0.05).

The efficiency of solely ciprofloxacin and gentamicin against cells in biofilms was low, apparently because of the protective properties of the biofilm (Figure 6, compare control and black bars). Benzalkonium chloride was able to reduce CFUs amount by 2 orders of magnitude. As can be seen from (Figure 6a,b), the combination of all antimicrobials with enzyme immobilized on medium molecular weight chitosan (bars labeled as PCh) led to a significant decrease in the number of CFUs (by approximately 3–4 orders of magnitude) in biofilms of both *Staphylococci*, similar to the samples treated with soluble papain (bars labeled by P). Of note, a significantly pronounced drop of viable cell count was observed when combining benzalkonium chloride with immobilized papain. Unexpectedly, the combination of all antimicrobials with papain immobilized on high molecular weight chitosan did not enhance the efficiency of antimicrobials against biofilm-embedded *S. aureus* (Figure 6c), while the anti-biofilm effect could be observed for ciprofloxacin and gentamicin against *S. epidermidis* even with pure chitosan (Figure 6d). Apparently, this effect could be attributed to the mechanical removal of the biofilm by high molecular weight chitosan particles.

## 3. Discussion

Papain, a non-specific sulfhydryl protease from the latex of *Carica papaya*, was intensively investigated as a promising enzyme for the food industry (for meat tenderization and production of protein hydrolysates), biotechnology (for bioactive peptides production), as well as medicine (as an anti-inflammatory, wound debriding, and healing agent) [52,53]. While effectively degrading necrotic tissues, papain does not affect healthy tissues due to its inactivation by α1-antitrypsin, which makes papain an attractive wound healing agent. Thus, in several studies papain-containing gels were reported as effective agents for venous ulcers healing, which can be safely used in granulation tissue [54,55,56].

Here we report the immobilization of papain on both medium and high molecular weight chitosan, as well as anti-biofilm properties of the immobilized enzyme. Chitosans, being natural polymers, exhibit broad-spectrum antimicrobial properties and are characterized by low toxicity on the one hand [57,58], while having great potential as an enzyme immobilization carrier on the other hand [59,60,61]. The developed approach for the adsorption-based immobilization of papain on matrixes of either medium or high molecular weight chitosans allowed coupling of up to 30% of the total protein (mg g chitosan^−1^) and specific activity (U mg protein^−1^), as well as above 90% of the initial total activity (U mL^−1^) measured by using azocasein as a substrate (see Table 1 and Table 2). Of note, the maximal adsorption of papain could be achieved at pH 9.0. Under these conditions, chitosan loses the charge [62], suggesting that the mechanism of immobilization is rather governed by hydrophobic adsorption than by ionic interactions. Nevertheless, the particular underlying mechanism is still not completely clear and requires further detailed investigations. 

The conformational changes observed during substrate–enzyme interactions and required for the catalytic act are determined by both preexisting conformations (conformational selection), as well as self-induced changes of the substrate on the enzyme conformation (induced fit) [63,64]. By contrast, immobilization increases the structural rigidity of the enzyme with the formation of more enzyme-support stable bonds. While the spacer arms are short enough and the support is rigid [65,66], this in turn leads to lower flexibility of the protein chain and consequently lower catalytic efficiency and specificity [63]. Immobilization on chitosans did not affect the K_m_ of papain, while V_max_ decreased 3-fold (Table 3, Figure 3), apparently, because of the limited flexibility of the enzyme molecule fixed on the surface of chitosan. Similar explanation could be proposed for the increased thermostability of the immobilized enzyme. Thus, the immobilized papain lost only one-half of its initial activity at 90 °C, while the soluble enzyme was completely inactivated (Figure 2). Next, while being stored in a buffer at 37 °C, the activity of pure enzyme was inactivated by 50% in 3.3 days, while immobilized papain maintained 83% of its initial activity even after 7 days (Figure 2a) with an extrapolated half-inactivation time of about 22 days for the medium and 18 days for the high molecular weight chitosans, respectively, under conditions tested. 

The obvious disadvantage of the adsorption-based immobilization could be fast desorption of the enzyme in solutions with even low ionic strength. Thus, in 0.9% NaCl half of the protein detached from the carrier in 24 h (Figure 2c). From the other side, this property could be used for wound-dressing materials design with dosed release of the enzyme to overcome relatively high cytotoxicity of the soluble papain, similar to enzyme loading into nanospheres, since the CC_50_ of papain has been reported to range from 6 to 260 µg mL^−1^ on various cell lines [12,67,68,69].

Besides wound debridement and healing applications, papain-gel with a red-light absorbing pigment (methylene blue–MB) has been proposed for the eradication of *Streptococcus mutans* biofilms [70]. The biofilm formation on chronic and acute wounds is an important factor that strongly affects their healing and prevents wound closure [71]. Being in the biofilm, bacteria are embedded into a self-produced extracellular matrix of organic polymers [72,73] that drastically reduces their susceptibility to antimicrobial treatment [74,75]. Our results indicate that papain itself efficiently degrades the structural components of the biofilm matrixes formed by *S. aureus, S. epidermidis, M. luteus*, including a substantial fraction of proteins [76]. In turn, being added at the same concentrations of 1 mg mL^−1^, papain led to a deeper destruction of the biofilms compared with trypsin, a protease that is widely used in wound treatment [50], although papain was less efficient than ficin (Figure 4). By contrast, only a weak effect of all proteases was observed against biofilms formed by *Pseudomonas aeruginosa, Escherichia coli*, and *B. cereus* (see Figure 4), apparently because their biofilm matrixes consist mainly of eDNA and polysaccharides [77]. Unfortunately, artificially high values were obtained in crystal violet staining of biofilms treated with high concentration of chitosans (solely or with immobilized papain) (Figure 5), while the photographs of plate bottoms treated with immobilized enzyme lacked the blue color corresponding to the biofilm stain (compare treated and control wells), suggesting removal of the biofilm. Less pronounced decrease of the blue stain was also observed in the wells treated solely by chitosan, apparently because of the mechanical scratching of the biofilm. Indeed, both medium and high molecular weight chitosans (solely or immobilized with papain) at concentrations of 14 and 17 mg mL^−1^, respectively, decreased the CFUs number of *Staphylococci* in the biofilm 10-fold (Figure 6), while the soluble enzyme led to a less pronounced drop of the adherent cells count. By contrast, only soluble papain and enzyme immobilized on medium molecular weight chitosan potentiated the efficacy of antimicrobials against biofilm-embedded *S. aureus* cells (Figure 6a,c). No statistically significant differences were observed between samples treated with any of the tested antimicrobial either in the presence or in the absence of either chitosans or papain immobilized on high molecular weight chitosan, suggesting the leading role of the enzymatic degradation of the biofilm in comparison with the mechanical destruction of *S. aureus* biofilms. Interestingly, this effect was not relevant for *S. epidermidis*, where the mechanical destruction of the biofilm also increased the efficacy of antimicrobials against biofilm embedded cells, although the combination of enzyme immobilized on medium molecular weight chitosan with either gentamicin or benzalkonium chloride led to almost complete eradication of the biofilm. This effect could be due to the differences in the biofilm structures formed by *S. aureus* and *S. epidermidis* and, consequently, their different permeabilities [78,79].

Thus, papain, either soluble or immobilized on medium molecular weight chitosan appears to be a beneficial agent for outer wound treatment capable of biofilms destruction, increasing the efficacy of antimicrobial treatment. The particular wound healing activity of both soluble and immobilized enzymes requires further investigation.

## 4. Conclusions

Thus, papain, either soluble or immobilized on medium molecular weight chitosan, efficiently destroys biofilms formed by *S. aureus* and *S. epidermidis* and potentiates the efficacy of antimicrobial treatment of biofilm-embedded *Staphylococci*. Moreover, while the adsorption-based immobilization of papain on chitosan leads to fast desorption of the enzyme in solutions, this property could be beneficial for wound-dressing materials design with dosed release of the enzyme to overcome relatively high cytotoxicity of the soluble papain. Taken together, our data indicate that papain immobilized on medium molecular weight chitosan appears a beneficial agent for outer wound treatment capable of the biofilms destruction, increasing the efficacy of wound treatment.

## 5. Materials and Methods

### 5.1. Chemicals

Papain was purchased from Sigma (P4762). Acid-soluble medium molecular (MMC, Mr = 200 kDa) and high molecular (HMC, Mr = 350 kDa) weight chitosans (Bioprogress, Shchelkovo Moscow region, Russia) were used as carriers for immobilization. Other chemicals were reagent grade and purchased from Sigma.

### 5.2. Papain Immobilization on Chitosans

The immobilization of papain on chitosans was performed using the adsorption approach developed previously for other proteases [80,81]. Briefly, chitosan (1 g) was hydrated by 24 h incubation in pure water, washed three times in water, and transferred into 20 mL of papain solution (1 mg mL^−1^) in various buffers as indicated in Table 1 and Table 2. The incubation was followed at 25 °C with stirring for 4 or 5 h for medium or high molecular weight chitosan, respectively. Next the chitosan was washed three times with the same buffer by decanting and dried overnight at 25 °C.

### 5.3. Proteolytic Activity Measurements

The proteolytic activity was evaluated by measuring the absorbance at 410 nm of fragments of proteolytic digestion of azocasein (Sigma-Aldrich, St. Louis, MO, USA), as described previously [82], with modifications [49].

### 5.4. Kinetic Properties

The enzymatic constants (K_m_,V_max_) of free and chitosan-immobilized papain were calculated according to the Michaelis–Menden curve and Lineweaver–Burk double reciprocal models by carrying out the enzymatic assay on azocasein (0.1–100.0 µM) in 50 mM Tris-HCl buffer (pH 7.5). The apparent K_m_ and V_max_ values were calculated. The enzyme turnover number k_cat_ was calculated from the Hanes–Woolf plot ([S]/v~[S] plot). 

### 5.5. Bacterial Strains and Growth Conditions

*Pseudomonas aeruginosa* (ATCC 27853), *Escherichia coli* (MG 1655), *Bacillus cereus* (clinical isolate), *Micrococcus luteus* (clinical isolate), *Staphylococcus aureus subsp. aureus* (ATCC 29213), and *Staphylococcus epidermidis* (clinical isolate) were used for the biofilm assays. Clinical isolates were obtained from Kazan Institute of Epidemiology and Microbiology (Kazan, Russia). Bacterial strains were maintained and grown on the LB medium. The Basal medium (BM) (glucose 5 g, peptone 7 g, MgSO_4_•7 H_2_O 2.0 g and CaCl_2_•2 H_2_O 0.05 g in 1.0 L tap water) was used for the biofilm formation assays [83,84]. To obtain rigid biofilms, bacteria were grown for 48 h under static conditions at 37 °C [84]. 

### 5.6. Determination of Minimum Inhibitory Concentration (MIC)

The MIC of antimicrobials was determined by the broth microdilution method in 96-well microtiter plates (Eppendorf) according to the EUCAST recommendations [85]. Briefly, the bacterial suspension containing 10^8^ CFUs mL^−1^ was subsequently diluted 1:300 with BM broth in microwell plates to obtain a 10^6^ cells mL^−1^ suspension and incubated at 37 °C for 24 h. Antimicrobials were added in the concentration range of 0.25–512 mg L^−1^. The MIC was determined as the lowest concentration of antimicrobial providing no visible bacterial growth after 24 h of incubation. To determine the MBC, 10 µL of culture liquid from wells without visible growth was seeded into 1 mL of fresh broth and incubated at 37 °C for 24 h. The antibiotic concentration corresponding to the absence of growth was considered as MBC.

### 5.7. Biofilm Assays

The bacterial biofilms were grown 48 h in BM broth in 24-well TC-treated polystyrol plates (1 mL per well). Then, the broth was exchanged with fresh broth supplemented with either soluble or chitosan-immobilized papain. After 24 h incubation, the plates were subjected to crystal violet staining [86]. To evaluate the potentiation of antimicrobials efficiency against biofilm-embedded cells by combination with enzymatic treatment, ciprofloxacin, gentamycin, or benzalkonium chloride was added at their respective 8× MBCs together with either soluble or chitosan-immobilized papain at the total protein concentration of 100 µg mL^−1^. After 24 h of incubation, the wells were analyzed by CFUs counting by drop plate assay [87] with modifications [88] using in-house developed software [89]. 

### 5.8. Statistical Analysis

Experiments were carried out in biological triplicates (i.e., newly prepared cultures and medium) with three technical repeats in each one. The statistical significance of results was assessed using the Kruskal–Wallis statistical test with significance threshold at *p* < 0.05. The enzymes half-inactivation time was calculated by plotting Log_10_(time) vs. percentage of residual enzyme activity in GraphPad Prism 6 (GraphPad Software, San Diego, CA, USA, www.graphpad.com, access date 11 March 2021).

## Figures and Tables

**Table 1:** The effect of buffers on papain adsorption on medium molecular weight chitosan from solution. In brackets, bound total protein, total and specific activities are shown as the percentage of their initial values, respectively.

Buffer System	pH	Total Protein, mg g Chitosan^−1^		Protein, mg mL^−1^	Activity, U mL^−1^		Specific Activity, U mg Protein^−1^	
0.2 M Sodium acetate	4.0	2.6 ± 0.50	(13.2%)	0.13 ± 0.02	42 ± 4.1	(44.6%)	320 ± 30.8	(33.8%)
	4.5	3.3 ± 0.38	(16.4%)	0.16 ± 0.02	79 ± 5.4	(83.7%)	484 ± 33.0	(51.0%)
	5.0	3.9 ± 0.37	(19.4%)	0.19 ± 0.02	82 ± 6.0	(87.0%)	425 ± 30.8	(44.9%)
	5.8	5.4 ± 0.84	(26.8%)	0.27 ± 0.04	90 ± 10.1	(95.4%)	337 ±37.7	(35.6%)
0.1 M di-potassium hydrogen phosphate	5.8	5.4 ± 0.51	(26.9%)	0.27 ± 0.03	22 ± 1.8	(23.5%)	83 ± 6.8	(8.7%)
	6.0	5.1 ± 0.40	(25.8%)	0.26 ± 0.02	18 ± 0.4	(20.0%)	73 ± 1.80	(7.8%)
	6.5	5.2 ± 0.22	(26.0%)	0.26 ± 0.01	23 ± 0.4	(24.6%)	89 ± 1.5	(9.4%)
	7.0	4.6 ± 0.45	(22.8%)	0.23 ± 0.02	18 ± 0.8	(19.6%)	81 ± 3.7	(8.6%)
	7.5	4.9 ± 0.28	(24.5%)	0.24 ± 0.01	17 ± 0.4	(18.6%)	72 ± 1.8	(7.6%)
	8.0	4.6 ± 0.28	(22.8%)	0.23 ± 0.01	19 ± 1.0	(20.5%)	85± 4.3	(9.0%)
0.05 M Tri-sodium borate, 0.1 M KCl	8.0	7.6 ± 0.76	(38.0%)	0.38 ± 0.04	38 ± 4.6	(40.6%)	101 ± 8.8	(10.7%)
	8.5	7.2 ± 1.04	(36.1%)	0.36 ± 0.05	39 ± 2.8	(41.4%)	109 ± 6.7	(11.5%)
	9.0	7.3 ± 0.79	(36.7%)	0.37 ± 0.04	39 ± 1.0	(41.7%)	108 ± 2.6	(11.4%)
	9.5	7.4 ± 0.89	(36.9%)	0.37 ± 0.04	39 ± 0.4	(41.2%)	106 ± 1.1	(11.1%)
	10.0	6.6 ± 0.41	(33.2%)	0.33 ± 0.02	41 ± 6.6	(43.0%)	123 ± 19.5	(12.9%)
0.05 M Tris-glycine	8.5	5.9 ± 0.24	(29.5%)	0.29 ± 0.01	82 ± 5.1	(86.7%)	275 ± 17.1	(29.0%)
	9.0	5.5 ± 0.17	(27.7%)	0.28 ± 0.01	88 ± 6.5	(92.7%)	317 ± 23.6	(33.4%)
0.05 M Glycine	8.6	5.8 ± 0.31	(28.8%)	0.29 ± 0.01	91 ± 2.0	(96.3%)	317 ± 6.2	(33.4%)
	9.0	5.9 ± 0.22	(29.6%)	0.30 ± 0.01	94 ± 2.3	(99.3%)	318 ± 24.9	(33.5%)
	9.5	5.9 ± 0.20	(29.5%)	0.29 ± 0.01	89 ± 2.7	(94.4%)	303 ± 7.9	(32.0%)
	10.0	5.8 ± 0.46	(29.3%)	0.29 ± 0.02	88 ± 3.8	(92.6%)	300 ± 12.9	(31.6%)
	10.5	5.7 ± 0.41	(28.5%)	0.28 ± 0.02	86 ± 1.9	(90.3%)	300 ± 5.8	(31.7%)

**Table 2:** The effect of buffers on papain adsorption on high molecular weight chitosan from solution. In brackets, bound total protein, total and specific activities are shown as the percentage of their initial values, respectively.

Buffer System	pH	Total Protein, mg g Chitosan^−1^		Protein, mg mL^−1^	Activity, U mL^−1^		Specific Activity, U mg Protein^−1^	
0.2 M Sodium acetate	4.0	3.4 ± 0.63	(16.8%)	0.17 ± 0.03	46 ± 8.8	(48.0%)	271 ± 52.3	(28.6%)
	4.5	4.7 ± 0.33	(21.3%)	0.21 ± 0.02	50 ± 8.4	(52.8%)	235 ± 39.5	(24.8%)
	5.0	6.9 ± 0.69	(34.4%)	0.34 ± 0.04	88 ± 4.6	(93.4%)	257 ± 13.3	(27.1%)
	5.8	7.1 ± 0.89	(35.7%)	0.36 ±0.05	78 ± 8.4	(82.0%)	218 ± 23.7	(23.0%)
0.1 M di-potassium hydrogen phosphate	5.8	5.7 ± 0.64	(28.3%)	0.28 ±0.03	26 ± 2.4	(27.7%)	92 ± 5.3	(9.8%)
	6.0	5.3 ± 0.54	(26.5%)	0.26 ± 0.03	27 ± 2.0	(28.5%)	102 ± 7.6	(10.7%)
	6.5	5.0 ± 0.33	(25.4%)	0.25 ± 0.01	27 ± 2.1	(28.6%)	107 ± 6.7	(11.3%)
	7.0	4.7 ± 0.74	(23.4%)	0.23 ± 0.02	25 ± 1.8	(26.9%)	109 ± 5.6	(11.6%)
	7.5	4.3 ± 0.47	(21.4%)	0.21 ± 0.02	25 ± 1.4	(26.4%)	117 ± 8.2	(12.3%)
	8.0	4.2 ± 0.35	(21.1%)	0.21 ± 0.01	24 ± 1.3	(25.7%)	114 ± 10.0	(12.0%)
0.05 M Tri-sodium borate, 0.1 M KCl	8.0	8.2 ± 0.68	(41.3%)	0.41 ± 0.03	94 ± 5.1	(99.6%)	229 ± 8.3	(24.1%)
	8.5	7.9 ± 1.19	(39.7%)	0.40 ± 0.06	93 ± 6.4	(98.7%)	235 ± 16.1	(24.8%)
	9.0	7.5 ± 0.73	(37.6%)	0.38 ± 0.04	93 ± 3.8	(98.8%)	249 ± 10.2	(26.3%)
	9.5	6.2 ± 1.91	(30.1%)	0.31 ± 0.09	94 ± 3.2	(99.6%)	305 ± 10.4	(21.2%)
	10.0	5.9 ± 1.08	(29.4%)	0.29 ± 0.05	94 ± 5.3	(99.1%)	319 ± 10.1	(33.7%)
0.05 M Tris-glycine	8.5	8.4 ± 1.5	(42.2%)	0.42 ± 0.08	92 ± 6.9	(97.4%)	219 ± 16.3	(23.1%)
	9.0	7.2 ± 0.8	(36.2%)	0.36 ± 0.04	91 ± 5.2	(96.6%)	253 ± 14.3	(26.7%)
0.05 M Glycine	8.6	7.0 ± 0.6	(35.1%)	0.35 ± 0.03	86 ± 5.2	(91.1%)	246 ± 10.4	(26.0%)
	9.0	7.2 ± 1.04	(36.0%)	0.36 ± 0.05	94 ± 7.9	(99.8%)	263 ± 15.3	(27.7%)
	9.5	8.2 ± 0.47	(40.9%)	0.41 ± 0.02	94 ± 4.2	(99.5%)	231 ± 9.6	(24.3%)
	10.0	8.1 ± 0.9	(40.7%)	0.41 ± 0.05	94 ± 5.7	(99.0%)	230 ± 11.7	(24.3%)
	10.5	7.9 ± 1.2	(39.7%)	0.39 ± 0.06	93 ± 9.7	(98.3%)	235 ± 24.4	(24.8%)

**Table 3:** Kinetic constants of soluble and immobilized papain.

Sample	K_m_, µM	V_max_, µM mg^−1^ min^−1^	k_cat_, min^−1^	V_max_/K_m_	k_cat_/K_m_
Soluble papain	22.3 ± 3.5	1253 ± 64	57.8 ± 2.9	56.2	2.59
Medium molecular weight chitosan immobilized papain	24.7 ± 5.4	432 ± 32	6.8 ± 0.5	17.3	0.28
High molecular weight chitosan immobilized papain	23.1 ± 4.3	349 ± 21	4.5 ± 0.3	15.2	0.19

**Table 4:** Minimum Inhibitory Concentration (MIC) and Minimal Bactericidal Concentrations (MBC) values of antimicrobials.

	*S. aureus*		*S. epidermidis*	
	MIC, µg mL^−1^	MBC, µg mL^−1^	MIC, µg mL^−1^	MBC, µg mL^−1^
Gentamycin	4	16	1	4
Ciprofloxacin	2	32	0.5	4
Benzalkonium chloride	1	16	1	4

## Data Availability

The data presented in this study are available on request from the corresponding author.
